# Role of Serotonin (5-HT) in GDM Prediction Considering Islet and Liver Interplay in Prediabetic Mice during Gestation

**DOI:** 10.3390/ijms23126434

**Published:** 2022-06-09

**Authors:** Melissa Asuaje Pfeifer, Moritz Liebmann, Till Beuerle, Katharina Grupe, Stephan Scherneck

**Affiliations:** 1 Institute of Pharmacology, Toxicology and Clinical Pharmacy, Technische Universität Braunschweig, Mendelssohnstraße 1, D-38106 Braunschweig, Germany; melissa.asuaje-pfeifer@tu-braunschweig.de (M.A.P.); m.liebmann@tu-braunschweig.de (M.L.); k.grupe@tu-braunschweig.de (K.G.); 2 Institute of Pharmaceutical Biology, Technische Universität Braunschweig, Mendelssohnstraße 1, D-38106 Braunschweig, Germany; t.beuerle@tu-braunschweig.de

**Keywords:** serotonin, prediabetes, gestational diabetes, GDM subtypes, islets of Langerhans, liver

## Abstract

Gestational diabetes (GDM) is characterized by a glucose tolerance disorder. This may first appear during pregnancy or pre-exist before conception as a form of prediabetes, but there are few data on the pathogenesis of the latter subtype. Female New Zealand obese (NZO) mice serve as a model for this subpopulation of GDM. It was recently shown that GDM is associated with elevated urinary serotonin (5-hydroxytryptamine, 5-HT) levels, but the role of the biogenic amine in subpopulations with prediabetes remains unclear. 5-HT is synthesized in different tissues, including the islets of Langerhans during pregnancy. Furthermore, 5-HT receptors (HTRs) are expressed in tissues important for the regulation of glucose homeostasis, such as liver and pancreas. Interestingly, NZO mice showed elevated plasma and islet 5-HT concentrations as well as impaired glucose-stimulated 5-HT secretion. Incubation of isolated primary NZO islets with 5-HT revealed an inhibitory effect on insulin and glucagon secretion. In primary NZO hepatocytes, 5-HT aggravated hepatic glucose production (HGP), decreased glucose uptake (HGU), glycogen content, and modulated AKT activation as well as cyclic adenosine monophosphate (cAMP) increase, indicating 5-HT downstream modulation. Treatment with an HTR2B antagonist reduced this 5-HT-mediated deterioration of the metabolic state. With its strong effect on glucose metabolism, these data indicate that 5-HT is already a potential indicator of GDM before conception in mice.

## 1. Introduction

GDM is a complex metabolic disease caused by inadequate adaptation of β-cells to compensate for the increased maternal insulin demand that occurs during pregnancy. Nevertheless, apart from this inability to compensate, the presence of insulin resistance (IR) itself is a risk factor and plays an important role in the development of GDM [1]. Adaptive mechanisms to compensate and to prevent hyperglycemia include an increase in both β-cell mass and insulin secretion [2,3]. The disease is commonly diagnosed with an oral glucose tolerance test (OGTT), which is performed in the second or third trimester of pregnancy and represents the current gold standard for diagnosing GDM [4]. Due to its heterogeneity, Powe et al. defined subtypes of GDM depending on whether an insulin secretion or insulin sensitivity defect predominates [5]. However, the current routinely used method of OGTT is not able to classify all subtypes and, moreover, there is no established method yet to diagnose GDM at an earlier stage of pregnancy in a standardized way. However, this could help to prevent serious disease-related outcomes for mother and child such as macrosomia, neonatal hypoglycemia, and perinatal death [6]. Therefore, various biomarkers for prediction of GDM have been discussed [7,8,9].

Recently, it has been shown that pregnant women with GDM have elevated urinary 5-HT levels compared to healthy pregnant women [10,11]. 5-HT is a monoamine which is synthesized in a two-step reaction from the amino acid tryptophan through tryptophan hydroxylase (TPH) and aromatic L-amino acid decarboxylase (AADC) [12]. About 95% of 5-HT is synthesized by enterochromaffin cells, subsequently taken up and stored by platelets. As platelets are activated, the biogenic amine is released and it is available in its free form for HTR binding [13]. During pregnancy, prolactin and placental lactogen (PL) signaling increases, leading to activation of both TPH isoforms TPH1 and TPH2 in pancreatic β-cells and subsequent 5-HT synthesis [14,15]. Lactogenic hormones are known to promote maternal β-cell proliferation, insulin synthesis, and glucose-stimulated insulin secretion during pregnancy, thereby protecting against the development of GDM. However, it should be noted that in mice there are two PLs called placental lactogen I (mPL-I) and mPL-II, while humans have a single human placental lactogen (hPL) [16,17]. Moreover, it could be shown that maternal 5-HT is crucial for normal fetal development [18]. In pancreatic islets, 5-HT enhances β-cell proliferation and mass expansion through HTR2B [15]. Recently, 5-HT secreted by β-cells has been shown to reduce glucagon secretion in human α-cells through activation of HTR1F, although its role in insulin secretion remains controversial [19]. A stimulating effect on insulin secretion was observed in pregnant and diet-induced insulin-resistant mice through HTR3 [20,21]. This is in contrast with observations showing that increased expression of HTR2C in pancreatic β-cells of db/db mice was associated with decreased insulin secretion [22]. Comparable effects could also be obtained in MIN6 cells which exhibited decreased insulin secretion after incubation with 5-HT mediated through HTR2B and HTR1A [23,24].

In addition to its effects in the islets of Langerhans, 5-HT has been shown to mediate important metabolic signals in other tissues. While 5-HT is not synthesized in the liver, in both humans and mice, hepatocytes express HTRs [25]. Of the more than 15 known receptors, mainly the HTR2 subtypes are expressed in the liver [26]. They are primarily thought to promote liver regeneration, as the expression of the major types HTR2A and HTR2B increases after hepatectomy [25]. It was demonstrated that 5-HT has significant effects by influencing hepatic gluconeogenesis, fatty acid metabolism, and liver regeneration [25,27,28]. These effects play a pivotal role in balancing energy homeostasis and have a direct impact on metabolic adverse conditions, like obesity and non-alcoholic fatty liver disease (NAFLD) [29]. Hepatic upregulation of HTR2s in particular has been associated with the latter [30]. Moreover, the inhibition of hepatic HTR2A signaling distinctly improved high-fat diet (HFD)-induced steatosis [31]. This further indicates that hepatic metabolism is highly affected by 5-HT through HTR2A activation. However, there are limited data on the influence and interaction of insulin and 5-HT. Therefore, it is of great significance to understand the complex interplay of secretion control of the islets of Langerhans and liver metabolism during pregnancy [32].

Female NZO mice serve as a model for prediabetes and represent subpopulations of human GDM by exhibiting impaired glucose tolerance (IGT) and a predominant insulin secretion defect [33]. Furthermore, NZO mice are characterized by preconceptional hyperinsulinemia and hyperglucagonemia that improves during gestation. Pregnant NZO mice showed a proliferation defect in islet cells and improved stimulability of insulin secretion during an OGTT, which was not observable in ex vivo perifusion experiments with isolated primary islets. However, the improved stimulability of insulin secretion detected in vivo was not sufficient to reverse IGT in NZO mice [33].

To date, there are few data on maternal serotonergic metabolism under prediabetic conditions, which incorporate islet and liver metabolism. Thus, the influence of 5-HT on this phenotype should be further investigated in NZO mice. The aim of this study was to elucidate the functional role of 5-HT in a prediabetic mouse model resembling a GDM-like phenotype, under consideration of the complexity of the multi-organ-affecting disease GDM. In isolated islets of Langerhans from NZO mice, it should be shown whether 5-HT affects insulin and glucagon secretion and therefore contributes to derangements in islet metabolism that may worsen the severity of the disease. We furthermore aimed to investigate whether 5-HT affects hepatic glucose metabolism, especially with regard to HGP and AKT activation as an indicator of insulin sensitivity. In addition, it should be clarified whether 5-HT could play a predictive role for the occurrence of GDM.

## 2. Results

### 2.1. Increased Plasma 5-HT Level and Islet 5-HT Content but Diminished Islet 5-HT Secretion in Female NZO Mice

**Figure 1:**
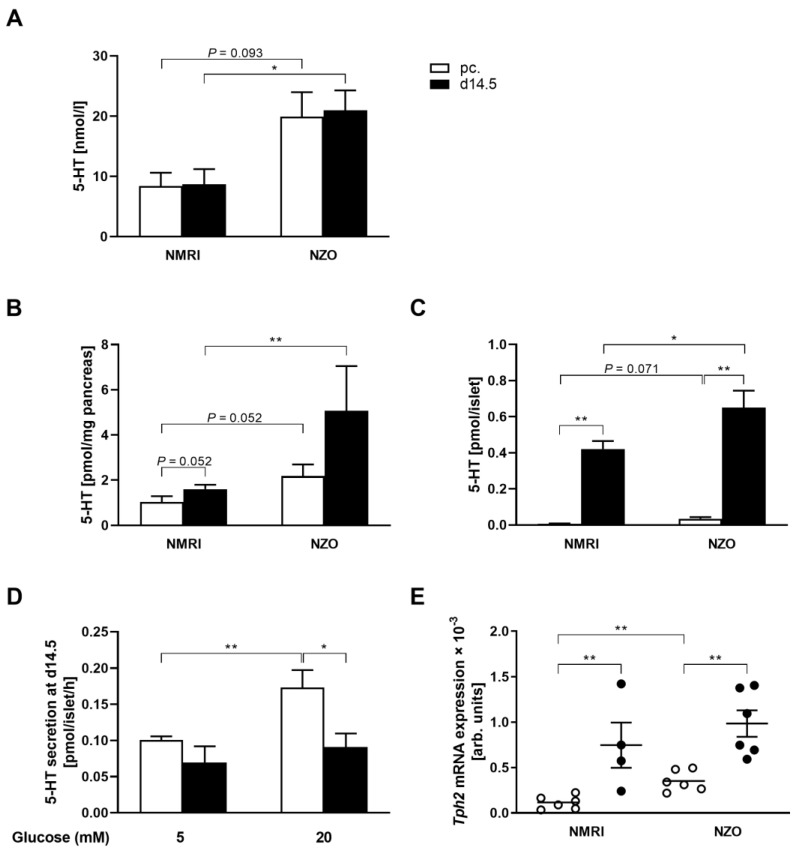
Elevated 5-HT plasma levels and increased 5-HT concentrations but impaired 5-HT secretion in islets of Langerhans of female NZO mice. (**A**) Random plasma 5-HT concentrations of NMRI and NZO mice at time points preconceptional (white bars) and d14.5 of gestation (black bars). (**B**) Total pancreatic 5-HT content and (**C**) islet 5-HT content were determined in acidic extracts preconceptionally (white bars) and at d14.5 of gestation (black bars) of NMRI and NZO mice. (**D**) 5-HT secretion after static incubation with 5 and 20 mM glucose at d14.5 of NMRI (white bars) and NZO mice (black bars). (**E**) *Tph2* gene expression in islets of NMRI and NZO mice at time points preconceptional (white circles) and d14.5 (black circles). Data are presented as means ± SEM (**A** *n* = 6, **B** *n* = 4–6, **C** *n* = 4–6, **D** *n* = 6, and **E** *n* = 4–6 animals per group). * *p* < 0.05; ** *p* < 0.01.

**Figure 2:**
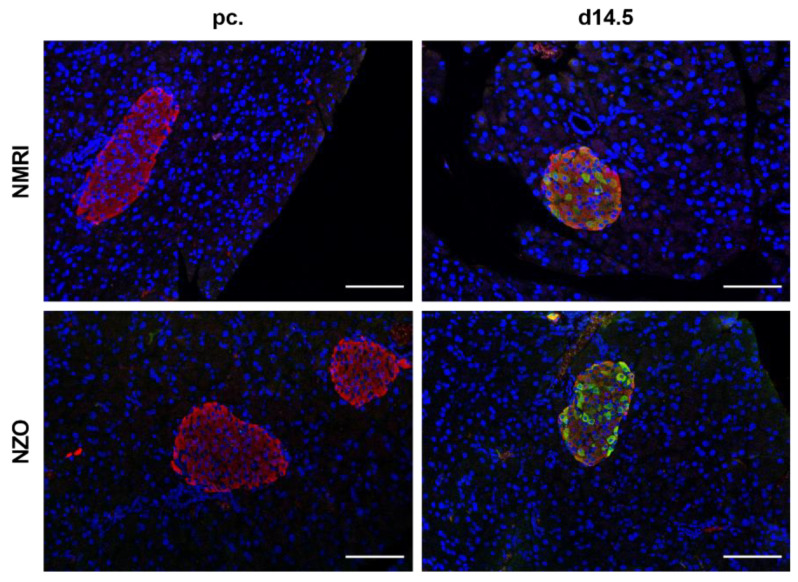
Co-localization of insulin and 5-HT in islets of Langerhans during gestation. Representative images of a double immunofluorescence staining for insulin (red) and 5-HT (green) at time points preconceptional and d14.5 of gestation. Nuclei were stained with DAPI (blue). Scale bars, 100 μm.

To determine differences in serotonergic metabolism, plasma 5-HT levels, total pancreatic 5-HT contents, islet 5-HT contents, and *Tph2* gene expression were examined preconceptionally and at day 14.5 (d14.5) of gestation. NZO mice showed twofold higher plasma 5-HT levels compared with NMRI mice. Significant difference between both strains was observed at d14.5 of gestation (NZO vs. NMRI, preconceptional: 19.92 ± 4.07 vs. 8.40 ± 2.21 nmol/L, *p* = 0.093; d14.5: 20.99 ± 3.30 vs. 8.71 ± 2.50 nmol/L, *p* < 0.05) (Figure 1A). Total pancreatic 5-HT content of NZO mice was slightly but not significantly increased preconceptionally, whereas a significant increase occurred at d14.5 of gestation (NZO vs. NMRI, preconceptional: 2.19 ± 0.51 vs. 1.04 ± 0.25 pmol/mg pancreas, *p* = 0.052; d14.5: 5.07 ± 1.98 vs. 1.60 ± 0.19 pmol/mg pancreas, *p* < 0.01). During gestation, NMRI (1.60 ± 0.19 vs. 1.04 ± 0.25 pmol/mg pancreas, *p* = 0.052) and NZO mice (5.07 ± 1.98 vs. 2.19 ± 0.51 pmol/mg pancreas, *p* = 0.114) showed a trend toward increased pancreatic 5-HT concentrations (Figure 1B). Islet 5-HT contents exhibited a similar distribution pattern at both time points in NZO mice, with a slightly but not significantly elevated 5-HT content preconceptionally and a significant increase at d14.5 of gestation (NZO vs. NMRI, preconceptional: 0.03 ± 0.01 vs. 0.01 ± 0.002 pmol/islet, *p* = 0.071; d14.5: 0.65 ± 0.09 vs. 0.42 ± 0.04 pmol/islet, *p* < 0.05). During gestation, a significant increase in islet 5-HT content was observed in NMRI (0.42 ± 0.04 vs. 0.01 ± 0.002 pmol/islet, *p* < 0.01) as well as in NZO islets (0.65 ± 0.09 vs. 0.03 ± 0.01 pmol/islet, *p* < 0.01) (Figure 1C). To estimate differences in stimulated 5-HT secretion, freshly isolated islets were incubated with 5 or 20 mM glucose at d14.5 of gestation. In NMRI mice, stimulation with 20 mM glucose resulted in a significant increase in 5-HT secretion compared with low glucose (0.17 ± 0.02 vs. 0.10 ± 0.005 pmol/islet/h, *p* < 0.01), while there was no substantial response in NZO mice. At low glucose concentrations, significant differences in 5-HT secretion could not be observed between the two strains (Figure 1D). To investigate possible differences in 5-HT synthesis, TPH2 was examined. *Tph2* gene expression increased significantly during gestation in NMRI (0.75 ± 0.25 vs. 0.12 ± 0.03, *p* ˂ 0.01) as well as in NZO islets (0.99 ± 0.15 vs. 0.35 ± 0.05, *p* ˂ 0.01). Gene expression was already elevated preconceptionally in NZO mice compared with the control (NZO vs. NMRI: 0.35 ± 0.05 vs. 0.12 ± 0.03, *p* ˂ 0.01) (Figure 1E). Representative immunofluorescence co-staining for insulin and 5-HT showed co-localization of insulin and 5-HT in islets of pregnant mice in both strains. Preconceptionally, neither NZO nor NMRI islets exhibited visible 5-HT staining (Figure 2). NZO mice exhibited increased 5-HT concentrations in plasma, pancreas, and islets as well as elevated 5-HT gene expression, while 5-HT secretion was glucose-independent in this model.

### 2.2. 5-HT Affected Both Insulin and Glucagon Secretion in NZO Islets

**Figure 3:**
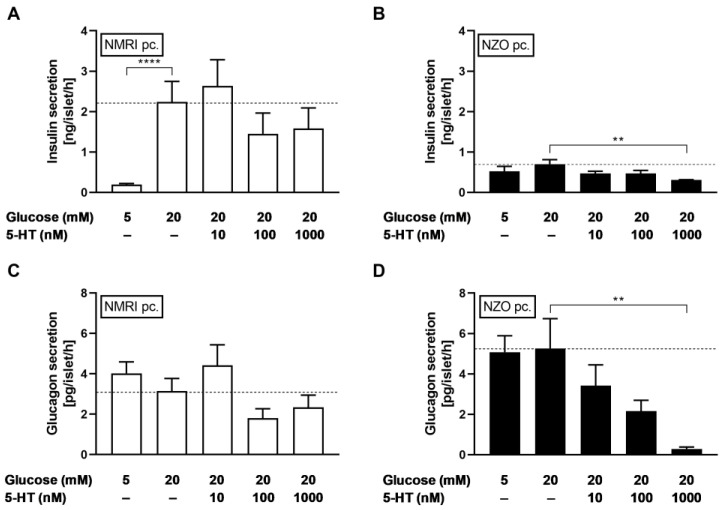
Decreased insulin and glucagon secretion in freshly isolated islets of Langerhans by 5-HT. Insulin (upper graphs) and glucagon secretion (lower graphs) of preconceptional (**A**,**C**) NMRI and (**B**,**D**) NZO islets after static incubation with 5, 20, and 20 mM glucose in combination with 5-HT. Data are presented as means ± SEM ((**A**) *n* = 8–9, (**B**) *n* = 7–8, (**C**) *n* = 8–9, and (**D**) *n* = 7–9 animals per group). ** *p* < 0.01; **** *p* < 0.0001.

**Figure 4:**
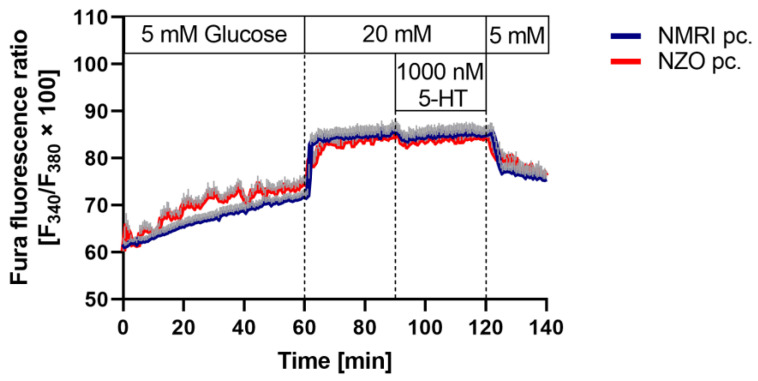
Deviating [Ca^2+^]_i_ in freshly isolated islets of Langerhans by 5-HT. [Ca^2+^]_i_ of preconceptional NMRI (blue line) and NZO (red line) mice. Islets were perifused from 0 min to 60 min with medium containing 5 mM glucose. Glucose concentration was increased to 20 mM glucose after 60 min and 1000 nM 5-HT was added after 90 min. This was followed by a 20 min washout period with 5 mM glucose. Data are presented as means ± SEM (*n* = 4 animals per group).

To examine the effects of elevated 5-HT levels on insulin and glucagon secretion in NZO mice during gestation, preconceptional islets were induced to a gestational-like state by incubation with 5-HT. Glucose-stimulated insulin secretion (GSIS) of isolated islets was inhibited significantly by high 5-HT concentration in NZO mice (1000 nM 5-HT + 20 mM glucose vs. 20 mM glucose: 0.31 ± 0.01 vs. 0.70 ± 0.11 ng/islet/h, *p* < 0.01) (Figure 3B). A comparative inhibitory effect after incubation with 1000 nM 5-HT was observed on glucagon secretion in NZO islets (1000 nM 5-HT + 20 mM glucose vs. 20 mM glucose: 0.29 ± 0.10 vs. 5.26 ± 1.47 pg/islet/h, *p* < 0.01) (Figure 3D). NMRI mice showed a trend toward inhibited insulin and glucagon secretion after incubation with high concentrations of 5-HT (Figure 3A,C). To investigate the effects of 5-HT on cytosolic Ca^2+^ concentration ([Ca^2+^]_i_), preconceptional islets were perifused with 5-HT. Addition of 1000 nM 5-HT to the 20 mM glucose stimulus decreased [Ca^2+^]_i_ in islets of both strains to an equal extent (Figure 4). In NZO mice, 5-HT led to an inhibition of insulin and glucagon secretion.

### 2.3. Effects of 5-HT on Hepatic Glucose Utilization in Primary Hepatocytes

**Figure 5:**
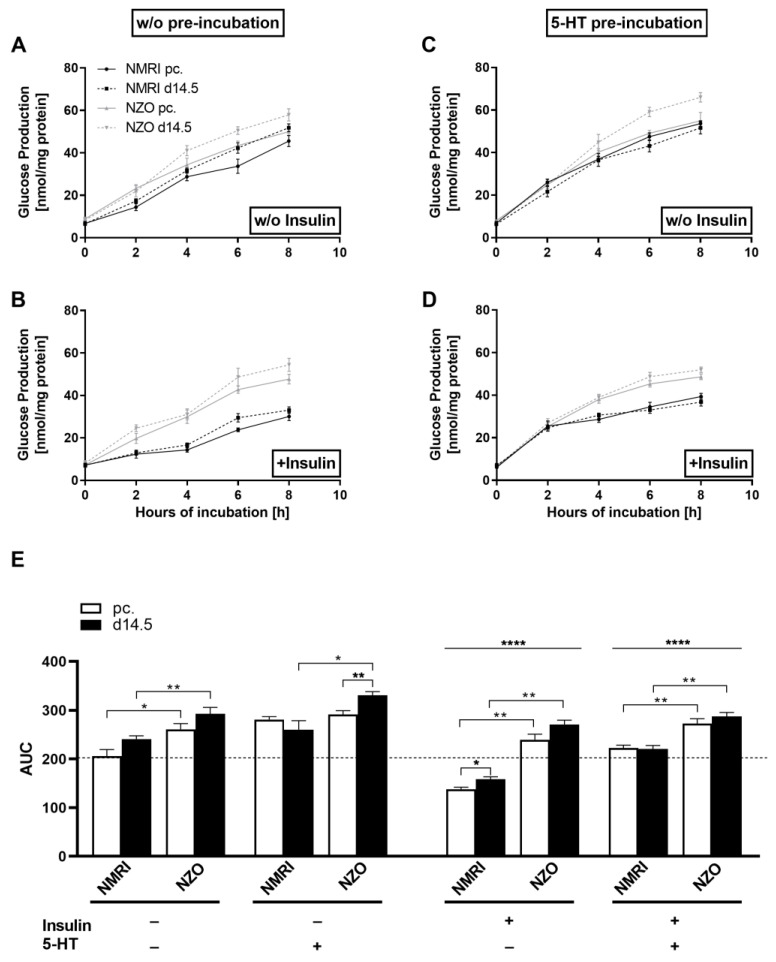
Altered glucose utilization in primary NZO hepatocytes. Glucose production in cultured primary hepatocytes of female NZO and NMRI control mice at time points preconceptional and d14.5 of gestation. Preincubation without (*w*/*o*) (**A**,**B**) or with 5-HT (**C**,**D**) and stimulation *w*/*o* (**A**,**C**) or with insulin (**B**,**D**). Area under the curve (AUC) for glucose production using the trapezoidal rule at time points preconceptional (white bars) and d14.5 (black bars) (**E**). Data are presented as means ± SEM (*n* = 5 animals per group). * *p* < 0.05, ** *p* < 0.01, multiple comparison tests results between groups: *w*/*o* I vs. +I: **** *p* < 0.0001, +I vs. +I+5-HT: **** *p* < 0.0001.

Due to the elevated 5-HT plasma levels in NZO mice compared to NMRI controls, the effect of the hormone on the hepatic glucose metabolism was investigated. HGP was determined in a co-stimulation setting, in which isolated primary hepatocytes of both strains were studied preconceptionally and at d14.5 of gestation. Without insulin as stimulant, HGP was increased in NZO primary hepatocytes at both time points compared to NMRI controls (NZO vs. NMRI, preconceptional: 261.03 ± 11.97 vs. 206.16 ± 13.31, *p* < 0.05; d14.5: 293.12 ± 13.00 vs. 241.11 ± 6.82 nmol/mg protein × 8 h, *p* < 0.05) (Figure 5A). A trend toward an increase in HGP over the period of time was observed in cells of both strains when comparing time point preconceptional with d14.5 of gestation. After insulin stimulation, HGP was significantly enhanced in NZO primary cells at both time points compared to the NMRI controls (NZO vs. NMRI, preconceptional: 239.94 ± 11.15 vs. 138.27 ± 3.79; *p* < 0.01; d14.5: 271.22 ± 8.73 vs. 158.57 ± 4.78 nmol/mg protein × 8 h, *p* < 0.01) (Figure 5B). Further, a significantly increased HGP in the NMRI cells at time point d14.5 compared to preconceptional was observed. This was probably due to the pregnancy-induced IR, which causes increased utilization (138.27 ± 3.79 vs. 158.57 ± 4.78 nmol/mg protein x 8 h, *p* < 0.05). To investigate effects of 5-HT on the HGP, preincubated cells of both strains were examined (Figure 5C). After 5-HT preincubation, an approximately 1.17-fold increase in HGP was observed in both strains at both time points. Further, HGP was significantly increased in NZO cells at time point d14.5 compared to preconceptional. (291.49 ± 7.97 vs. 330.71 ± 7.95 nmol/mg protein × 8 h, *p* < 0.01). Likewise, the HGP was significantly increased in NZO cells at d14.5 compared to NMRI controls (NZO vs. NMRI: 330.71 ± 7.95 vs. 260.63 ± 18.07 nmol/mg protein × 8 h, *p* < 0.05). Within the NMRI strain, no difference was detected. Co-stimulation with insulin counteracted the 5-HT effect in both strains. Nevertheless, NZO cells still exhibited significantly increased HGP after co-stimulation with 5-HT and insulin compared to the NMRI controls (NZO vs. NMRI: 272.85 ± 10.04 vs. 222.69 ± 5.69, *p* < 0.01; d14.5: 287.56 ± 8.22 vs. 220.41 ± 7.27 nmol/mg protein × 8 h, *p* < 0.01). There were no differences after co-stimulation within the strains at the times studied. In each treatment condition, the NZO primary cells showed an increased HGP compared to the NMRI controls, especially at d14.5. Further, the NZO cells displayed a decreased response to insulin as a stimulant, in both treatments, with and without prior 5-HT preincubation, which led to an elevated utilization compared to the NMRI controls (Figure 5E). Nevertheless, in relation to the treatment groups, cells stimulated solely with insulin showed significantly reduced HGP compared to those unstimulated (*p* < 0.0001). Notably, HGP in the 5-HT and insulin co-stimulated condition was significantly higher compared to the insulin-only-treated condition (*p* < 0.0001). In parallel, primary hepatocytes of both strains showed slightly elevated utilization compared to the unstimulated condition after the preincubation solely with 5-HT.

### 2.4. Effects of Selective 5-HT Receptor Antagonists on Hepatic Glucose Utilization in Primary Hepatocytes

**Figure 6:**
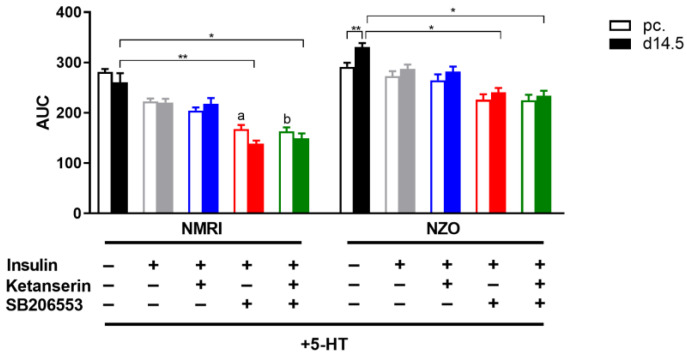
Effects of 5-HT on hepatic glucose production are mediated by 5-HTR2B/C. Area under the curve (AUC) for glucose production in cultured primary hepatocytes of NMRI and NZO mice using the trapezoidal rule at time points preconceptional (open bars) and d14.5 of gestation (filled bars) without or after initial insulin stimulus and incubation with 5-HT or co-incubation with HTR antagonists. Co-incubation with Ketanserin (HTR2A antagonist) and SB206553 (HTR2B/C antagonist). Data are presented as means ± SEM (*n* = 5 animals per group). * *p* < 0.05, ** *p* < 0.01, NMRI pc. *w*/*o* I vs. NMRI pc. +I+SB206553: ^a^ *p* < 0.05, NMRI pc. *w*/*o* I vs. NMRI pc. +I+Ketanserin+SB206553: ^b^ *p* < 0.01. For better illustration, data of 5-HT preincubation shown in Figure 5 were plotted again.

To further investigate the 5-HT effect on HGP, cells were stimulated with selective HTR antagonists (Figure 6 and Figure S1). While inhibition of HTR2A with Ketanserin had no effect on HGP compared to the 5-HT and insulin-stimulated control, treatment with HTR2B/C antagonist SB206553 inhibited the enhancing effect of 5-HT on the utilization. Further, primary cells of both strains showed significantly decreased HGP at d14.5 after SB206553 treatment compared to the 5-HT-only-stimulated control (NMRI: 260.63 ± 18.07 vs. 138.66 ± 6.26, *p* < 0.01; NZO: 330.71 ± 7.97 vs. 240.68 ± 8.67 nmol/mg protein × 8 h, *p* < 0.05). This was also observed preconceptionally in NMRI cells (281.31 + 6.02 vs. 167.37 + 8.36 nmol/mg protein × 8 h, *p* < 0.05). The co-administration of both inhibitors could verify the inhibitory effect of SB206553, indicating that treatment with HTR antagonists could selectively reverse the 5-HT effect. This demonstrated that the 5-HT effect is modulated via the HTR2B/C but not via the HTR2A.

### 2.5. Influence of 5-HT on Glucose Uptake and Glycogen Content in Primary Hepatocytes

**Figure 7:**
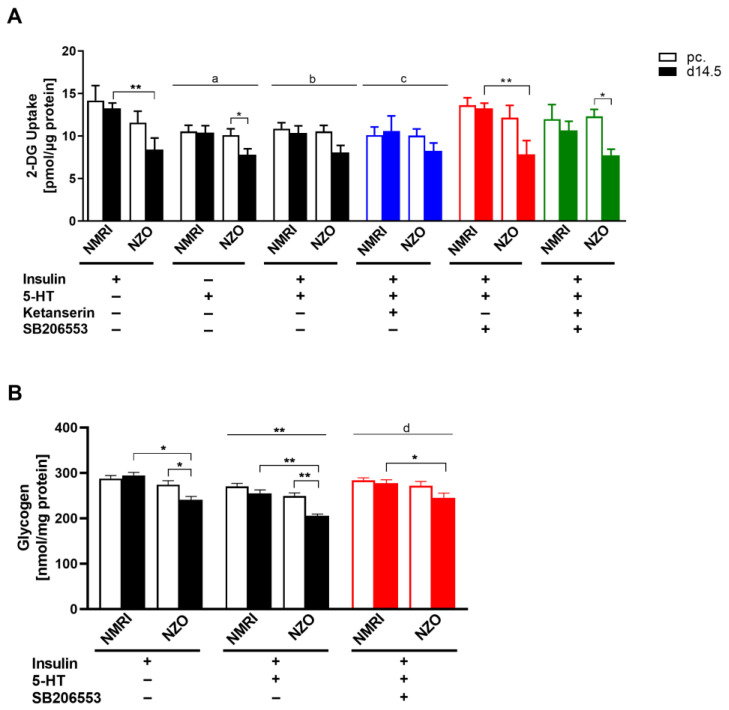
Impact of 5-HT on hepatic glucose metabolism in primary hepatocytes. (**A**) 2-DG uptake with insulin and 5-HT stimulation or co-incubation with 5-HTR antagonists. Co-incubation with Ketanserin (HTR2A antagonist) and SB206553 (HTR2B/C antagonist). (**B**) Glycogen content with insulin preincubation and 5-HT or SB206553 stimulation in cultured primary hepatocytes of NMRI and NZO mice at time points preconceptional (open bars) and d14.5 of gestation (filled bars). Data are presented as means ± SEM (**A** *n* = 4–6, **B** *n* = 5 animals per group). * *p* < 0.05, ** *p* < 0.01, multiple comparison tests results between groups: A +I vs. +5-HT: ^a^ *p* < 0.05, +I vs. +5-HT+I: ^b^ *p* = 0.063, +I vs. +5-HT+I+K: ^c^ *p* < 0.05, +I vs. +5-HT+I: ** *p* < 0.01; B +5-HT+I vs. +5-HT+I+SB206553: ^d^ *p* < 0.01.

Thereafter, we examined HGU and hepatic glycogen content as components of hepatic glucose homeostasis under ex vivo experimental conditions. To display a potential 5-HT effect, treatments with the HTR antagonists Ketanserin and SB206553 were included in both conducted experiments. After insulin stimulation, 2-deoxy-glucose (2-DG) uptake was significantly reduced at d14.5 in NZO hepatocytes compared to NMRI controls (NZO vs. NMRI: 8.43 ± 1.35 vs. 13.26 ± 0.63 pmol/μg protein, *p* < 0.01) (Figure 7A). The stimulation with 5-HT induced a decreased 2-DG uptake compared to the insulin-treated control in both strains (*p* < 0.05). After co-stimulation with 5-HT and insulin, 2-DG uptake could not be increased. Compared to the insulin-only stimulation, the 2-DG uptake modulating effect was no longer observed and uptake remained slightly reduced in both strains (*p* = 0.063). Co-administration with 5-HT and SB206533 could selectively reverse the 5-HT effect. In contrast, after Ketanserin and 5-HT co-administration, the 2-DG uptake remained significantly decreased (*p* < 0.05). Thus, the 5-HT effect is modulated by HTR2B/C but not by the HTR2A. Additionally, a 5-HT effect on glycogen content was observed (Figure 7B). After insulin stimulation, NZO cells exhibited significantly reduced glycogen content at d14.5 compared to NMRI controls (NZO vs. NMRI: 240.94 ± 7.40 vs. 294.94 ± 6.68 nmol/mg protein, *p* < 0.05). Within the NZO strain, primary cells at time point d14.5 showed a decreased glycogen content compared to preconceptional (274.50 ± 8.79 vs. 240.94 ± 7.40 nmol/mg protein, *p* < 0.05). Co-stimulation with 5-HT and insulin induced a significant decrease of glycogen content compared to the insulin-stimulated control (*p* < 0.01). However, despite the decreasing effect on glycogen content, NZO cells still displayed significantly less glycogen compared to NMRI controls at d14.5. Co-administration of 5-HT and insulin with SB206533 could selectively reverse the 5-HT effect. The glycogen content was significantly increased compared to the non-antagonized condition with 5-HT and insulin stimulation (*p* < 0.01). 

### 2.6. Effects of 5-HT on Hepatic cAMP and AKT Signaling

**Figure 8:**
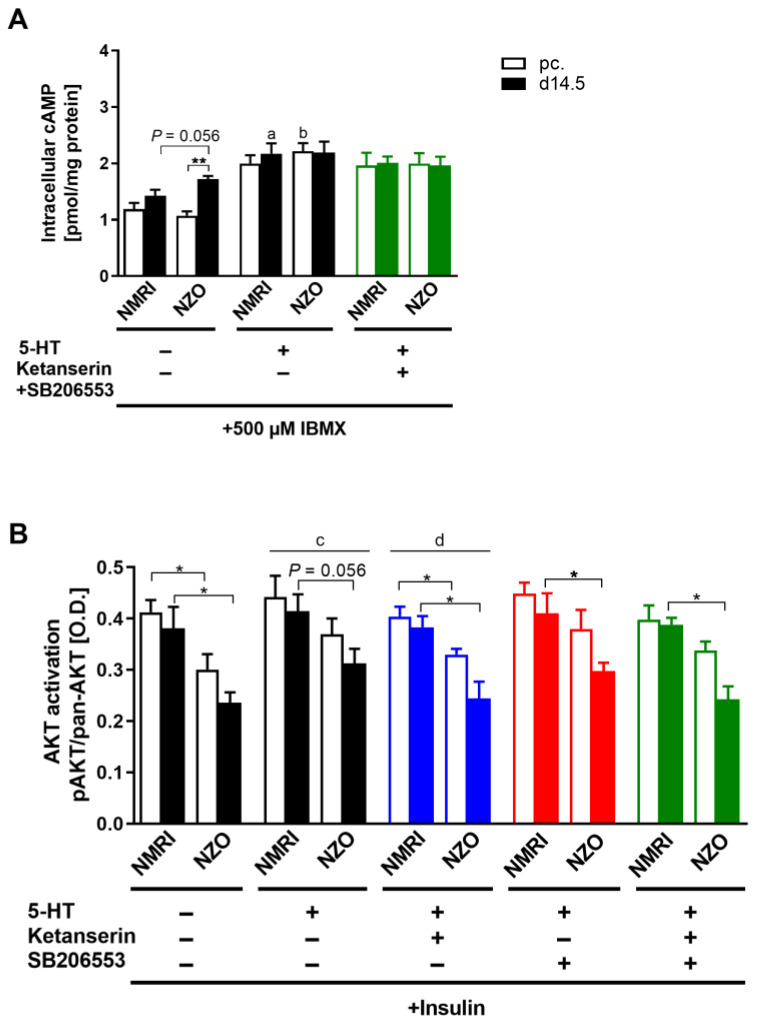
Impact of 5-HT on intracellular cAMP and AKT activation. (**A**) Intracellular cAMP content with 5-HT stimulation and co-incubation with Ketanserin and SB206553 in cultured primary hepatocytes of NMRI and NZO mice at time points preconceptional (open bars) and d14.5 of gestation (filled bars); IBMX, 3-Isobutyl-1-methylxanthine. (**B**) AKT-activation (pAKT/pan-AKT ratio) after initial insulin stimulation and incubation with 5-HT and co-incubation with Ketanserin (HTR2A antagonist) and SB206553 (HTR2B/C antagonist). Data are presented as means ± SEM (A *n* = 5, B *n* = 5–6 animals per group). * *p* < 0.01, ** *p* < 0.01, NMRI d14.5 vs. NMRI d14.5 +5-HT: ^a^ *p* < 0.05, NZO pc. vs. NZO pc. +5-HT: ^b^ *p* < 0.05, multiple comparison tests results between groups *w*/*o* 5-HT vs. +5-HT: ^c^ *p* < 0.05, +5-HT vs. +5-HT+Ketanserin: ^d^ *p* = 0.109.

To clarify the observed effects on glucose metabolism by 5-HT stimulation, hepatic glucose signaling was investigated. Unstimulated NZO primary cells already showed significantly increased intracellular cAMP content at time point d14.5 compared to preconceptional (1.07 ± 0.08 vs. 1.72 ± 0.06 pmol/mg protein, *p* < 0.01). Additionally, NZO cells exhibited a trend towards an increased content compared to unstimulated NMRI controls at d14.5 (NZO vs. NMRI: 1.72 ± 0.06 vs. 1.43 ± 0.11 pmol/mg protein, *p* = 0.056). After 5-HT stimulation, the cAMP contents were slightly increased in both strains at both time points. While the significant difference within the NZO cells, with increased cAMP levels at time point d14.5 compared to preconceptional was diminished, NMRI cells during gestation as well as preconceptional NZO cells exhibited significant increased cAMP contents compared to their unstimulated counterpart. However, the co-administration of both Ketanserin and SB206553 had no further effect on the cAMP contents. AKT activation was measured as the ratio of phosphorylated protein to pan-AKT (Figure S2) after initial insulin stimulation to induce phosphorylation (Figure 8B). Insulin-only-stimulated NZO cells exhibited significantly reduced AKT activation at both time points compared to NMRI controls (NZO vs. NMRI, preconceptional: 0.30 ± 0.03 vs. 0.41 ± 0.02, *p* < 0.05; d14.5: 0.24 ± 0.02 vs. 0.38 ± 0.04 O.D., *p* < 0.05). The stimulation with 5-HT increased AKT activation compared to the solely insulin-stimulated condition (*p <* 0.05). Moreover, the decreased AKT activation in the NZO cells remained detectable at d14.5. The treatment with HTR antagonists had no effect on AKT activation. 

## 3. Discussion

In the present work, we demonstrate changes in serotonergic metabolism under prediabetic conditions and during gestation in the presence of a GDM-like phenotype. Therefore, we used polygenic NZO mice, which were examined under the aspect of islet of Langerhans and liver glucose homeostasis and compared with the NMRI control strain. 5-HT concentrations were assessed in plasma, pancreas, and islet. Moreover, 5-HT secretion of the islet, *Tph2* gene expression, and the effect of the biogenic amine on insulin, glucagon secretion, and [Ca^2+^]_i_ of the islet were determined. Further, the impact of 5-HT on the hepatic glucose metabolism was investigated by the measurement of HGP, HGU, and hepatic glycogen as well as AKT activation and cAMP content. The data revealed a deteriorative effect of 5-HT on hepatic glucose homeostasis as well as on insulin secretion and affirmed its contribution to a prediabetic phenotype of the NZO mouse. The impact of 5-HT on the metabolism of both organs most likely makes it a suitable predictive indicator for GDM.

NZO mice exhibited elevated plasma 5-HT levels both preconceptionally and at d14.5 of gestation compared to the NMRI control strain. This has been previously described for rodents and humans with T2D, obesity, NAFLD, and following an HFD [31,34,35,36,37]. In addition, urine samples from pregnant women with GDM revealed elevated levels of 5-HT compared to women without GDM [10,11]. As already described for diabetic patients, these elevated free 5-HT levels may be due to an increased release of 5-HT from platelets combined with a lower uptake of 5-HT by platelets [34,35,38]. However, gestation showed no effect on plasma 5-HT levels in any of the two strains. Furthermore, it could be shown that total pancreatic and islet 5-HT contents were higher in NZO mice compared to NMRI mice at both time points. As expected, gestation resulted in elevated 5-HT content within the islets in both strains. This was confirmed by representative immunofluorescence staining, which further showed co-localization of insulin and 5-HT in islets of mice during gestation, suggesting that 5-HT is localized in the β-cells of both strains. The observed distribution pattern was largely consistent with changes in *Tph2* gene expression. The latter was increased preconceptionally in NZO mice compared with NMRI controls, although an elevation to comparable values was found in both strains during gestation. Since plasma prolactin levels did not differ between NZO and NMRI mice preconceptionally or at d14.5 of gestation, the increased 5-HT content in NZO islets cannot be explained by this lactogenic hormone and subsequent increased regulation of 5-HT biosynthesis [33]. However, it should be noted that prolactin secretion in rodents is suppressed during mid-gestation by an increase in placental lactogens [39]. Thus, an influence of placental lactogens would be conceivable here and should therefore be further investigated. Since 5-HT content is already preconceptionally elevated in NZO islets, this may also indicate pregnancy-independent reasons for the observed differences. Increased concentrations of 5-hydroxytryptophan (5-HTP) and its precursor tryptophan as well as decreased activity of the tryptophan-degrading enzyme indoleamine 2,3-dioxygenase (IDO) in β-cells might explain the elevated 5-HT content in NZO islets. However, it can be assumed that the increased 5-HT content during gestation in NZO islets is part of the compensatory mechanisms that should lead to increased β-cell mass in an autocrine/paracrine manner via the HTR2B [15]. Interestingly, recently published data from our group show a proliferation defect in NZO mice, suggesting that 5-HT has no effect on β-cell mass here [33].

5-HT has previously been shown to be co-secreted with insulin in a glucose-dependent manner, which is consistent with the findings on the 5-HT secretion pattern of the NMRI control during gestation [19,40]. In contrast to freshly isolated islets from NMRI mice, NZO islets did not show an adequate response to the glucose stimulus. This observation is in line with the impaired GSIS observed ex vivo at the time points preconceptional and d14.5 of gestation in NZO islets by our group [33]. Besides 5-HT, other neurotransmitters including γ-aminobutyric acid (GABA) are synthesized, stored, and secreted in pancreatic β-cells [41,42]. Similar to GABA, 5-HT might be co-localized not only with insulin in dense-core granules but also in synaptic microvesicles, which might explain the altered 5-HT secretion pattern and a possible glucose-independent 5-HT secretion in NZO islets [14,42,43].

Transferring preconceptionally isolated islets into a gestation-like state by incubation with high concentrations of 5-HT resulted in significant inhibition of insulin and glucagon secretion in NZO mice during stimulation with glucose. These observations are consistent with previous findings of other authors in MIN6 cells, rodents, and humans, although it should be noted that the influence of 5-HT on insulin secretion is still a matter of controversy [19,23,44,45,46,47]. An inhibitory effect on insulin secretion was also previously observed for the selective 5-HT re-uptake inhibitors (SSRIs) fluoxetine and sertraline in MIN6 cells as well as in mouse and human islets [48,49]. This is consistent with long-term use of SSRIs being associated with an increased risk of diabetes [50]. In addition, an inhibitory effect on [Ca^2+^]_i_ in preconceptional islets could be shown here in both strains when 5-HT was added to the perifusion medium containing stimulating glucose, supporting the observed inhibitory effect on insulin secretion. 

Recently, it has been shown that treatment of female mice with the second-generation antipsychotic (SGA) drug aripiprazole elevated *Tph1* and *Tph2* expression and that 5-HT content in islets increased to a level comparable to pregnant mice. Moreover, treatment with aripiprazole led to a doubling of β-cell mass and further ex vivo experiments with the SGA resulted in impaired insulin secretion [45]. These findings are in close agreement with those obtained here in prediabetic NZO mice and suggest that blockade of peripheral 5-HT synthesis might lead to an improvement of impaired GSIS and thus to a reduced risk of the development of GDM. In this regard, a study with HFD-induced diabetes in mice has already shown that oral administration of the TPH1 inhibitor LP533401 led to an improvement of peripheral insulin sensitivity and glucose tolerance as well as a reduction of hyperglycemia and hyperlipidemia [27]. It can be assumed that the observed effects of 5-HT on insulin and glucagon secretion may occur in an autocrine/paracrine manner and are most likely due to the inhibitory mechanisms of 5-HT already described for rodents and humans [19,22,24,51]. The increased 5-HT content in β-cells and the concomitant paracrine inhibition of α-cells by 5-HT may be the underlying reason for the improved hyperglucagonemia during pregnancy in NZO mice, which we previously reported [33].

Since 5-HT levels have already been associated with pathological changes in the liver, the hepatic metabolism of the NZO strain was investigated in more detail [36,37]. For instance, the use of SSRIs during pregnancy was linked to a higher risk of obesity and hyperlipidemia in rats [52]. Gut-derived 5-HT modulates adaptation to food intake and deprivation via gluconeogenesis and glucose uptake in the liver [27]. These effects show the diversity of 5-HT action and thus the broad range of effects on metabolism in target organs. Ming et al. used selective β-cell-selective *Sirt3* knockout (KO) mice and were able to show IGT and altered insulin secretion after HFD, which was accompanied by increased serum 5-HT level and 5-HT synthesis in islets and by upregulation of TPH1 [53]. In addition, enhanced HFD-induced hepatic steatosis was observed in this model. This was attributed to 5-HT promoting lipogenic metabolic pathways in primary mouse hepatocytes via HTR2A. Therefore, inhibition of 5-HT synthesis attenuated steatosis [53]. 

Prediabetic NZO mice revealed increased HGP unstimulated and even after insulin stimulation, which indicates hepatic IR, while co-stimulation with insulin counteracted the 5-HT-mediated response. Together with the significantly increased 5-HT plasma levels, a further increase and thus worsening of HGP in vivo can be assumed. This is in line with Sumara et al., who observed 5-HT-induced gluconeogenesis in primary hepatocytes [27]. Our results showed a receptor-specific effect of 5-HT, where treatment with HTR antagonists could selectively reverse the increasing 5-HT-mediated HGP. However, using the receptor-selective antagonists SB206553 and Ketanserin, we demonstrated that this effect is modulated via the HTR2B rather than the HTR2A. Although SB206553 is a potent antagonist of the HTR2C, this plays only a minor role in the liver, as it is involved in hepatic regeneration mediated by the autonomic nervous system [54,55]. This is consistent with Sumara et al. who demonstrated the promoting effect of 5-HT on hepatic gluconeogenesis via the HTR2B in mice [27]. As evidence, they showed attenuated HGP from pyruvate/lactate substrates when incubated with 5-HT in *Htr2b* KO mice. The implantation of 5-HT-releasing pellets confirmed the induction of HGP in vivo in mice with functional HTR2B. Moreover, the ability to induce HGP was confirmed in *Tph1* KO mice, which exhibited a significantly reduced production from endogenous substrates under conditions of hyperinsulinemic-euglycemic clamps. The action of 5-HT via both receptors demonstrates its importance for energy metabolism, whereby lipid metabolism is mediated via HTR2A, and glucose metabolism is mediated via HTR2B. 

After co-stimulation with insulin and 5-HT, HGU was slightly reduced in both strains compared to solely insulin stimulation. Co-stimulation with 5-HT and the SB206553 could selectively reverse the observed 5-HT effect. This demonstrated that the 5-HT effect on the HGU is modulated via the HTR2B/C, but not via HTR2A [27]. Although, an increasing effect of 5-HT on the glucose uptake was observed in isolated rat myocytes [56]. However, an underlying mechanism for the reduced HGU is still missing. 

Sodium-glucose-linked transporter 1 (SGLT1), necessary for intestinal glucose uptake, is known to be upregulated after intestinal glucose infusion and for triggering the upregulation of glucose transporter 2 (GLUT2) in the intestinal membrane [57,58]. The transporter is also expressed in the liver [59]. Further, there is limited evidence that SGLT1 is involved in 5-HT release. Therefore, it could possibly mediate glucose uptake over 5-HT via the cAMP/PKA signaling pathway as well [60,61,62]. Furthermore, Moore et al. reported increased HGU after 5-HT treatment, suggesting the potential of 5-HT to reduce postprandial hyperglycemia. However, these data were obtained from dogs, which calls into question their transferability to our model [63]. Watanabe et al. investigated the 5-HT effect on glucose uptake in different tissues to determine the mechanism by which 5-HT possibly elevates plasma glucose concentrations. However, the HGU was not affected in the liver [28]. Taken together, with the observed elevated HGP, the 5-HT-mediated effect on HGU may lead to an overall deterioration in glucose homeostasis. 

Impaired AKT activation is primarily associated with hepatic IR [64]. Despite AKT induction through 5-HT, the reduced AKT activation during gestation was maintained in NMRI mice, which mirrors the mildly induced physiologically acquired IR. The significantly increased IR in NZO is indicated by the strongly reduced AKT activation during gestation [65,66]. Treatment with HTR antagonists had no reversible effect on the AKT activation triggered by 5-HT. Further, Zhou et al. demonstrated with m-chlorophenylpiperazine (mCPP), a HTR2C agonist, significantly increased hepatic AKT activation in C57BL/6J DIO mice [67]. Taken together, 5-HT seems to modulate AKT activation directly over the phosphatidyl inositol 3-kinase (PI3K) pathway, and therefore does not have a primary IR-inducing effect. After 5-HT administration, cAMP levels were slightly increased. The treatment with SB206553 and Ketanserin had no further effect on intracellular cAMP content. However, in female Wistar rats, 5-HT administration stimulated the increase of cAMP content in the liver [68]. Therefore, serotonergic action in hepatocytes is in part mediated by an increase in intracellular cAMP, possibly mediated through HTR7s [69]. This signaling through HTR7 was successfully reversed by the HTR7 antagonist SB269970 [70].

5-HT treatment induced a significant reduction in glycogen content, although the administration of SB206553 could selectively reverse the 5-HT-mediated effect. Hampson et al. could demonstrate that 5-HT stimulated glycogen synthesis in short-term exposed rat primary hepatocytes. Interestingly, they observed a dose-dependent effect, whereby nanomolar concentrations stimulated synthesis and micromolar concentrations inhibited glycogen synthesis [71]. However, physiological 5-HT concentrations are in a nanomolar range [72]. In contrast, Levine et al. described glycogenolytic effects induced by 5-HT [73]. Noticeably, pregnant NZO mice showed reduced glycogen levels, which was not due to 5-HT, since the biogenic amine led neither to an improvement nor to a deterioration. Rather, as described by Irimia et al., this is likely due to the inability to synthesize glycogen in the liver, resulting in hepatic impairment of insulin signaling with reduced AKT response in mice [74]. Furthermore, reduced hepatic glycogen synthesis caused by lipid-induced hepatic IR was described [75].

In summary, our findings demonstrate the complexity and relevance of serotonergic metabolism to glucose homeostasis in pregnancy in both islets of Langerhans and liver. Our data show that 5-HT content is elevated in islets of prediabetic NZO mice and leads to inhibition of insulin and glucagon secretion. Furthermore, 5-HT had an aggravating effect on hepatic glucose metabolism in both the prediabetic and metabolically healthy strain. This becomes particularly evident in NZO mice due to the elevated plasma levels of 5-HT in vivo. Moreover, the elevated 5-HT levels found in women with GDM could be verified in our model. 

Moreover, we were able to show that elevated plasma 5-HT levels may be a biomarker for early prediction of GDM risk even before conception and are thus suitable as an additional diagnostic marker besides the established OGTT. In this way, a possible therapeutic intervention and monitoring in planned pregnancies can be started at an early stage. Therefore, the NZO mouse is suitable as a model for further research to investigate whether glucose metabolism is improved by interfering in 5-HT metabolism.

## 4. Materials and Methods

### 4.1. Animals

All procedures were performed under permits from the ethics committee of the Lower Saxony State Office for Consumer Protection and Food Safety (Oldenburg, Germany; ethics approval number: 33.19-42502-04-17/2462; internal IDs (05.15) TSB TU BS and (05.19) TSB TU BS). The NZO (NZO/HIBomDife) and the NMRI (NMRI/RjHan) control strain were used for this study. Due to its ordinary physiological adaptation to pregnancy and a known robust β-cell physiology, the NMRI strain was chosen as metabolically healthy control [33,76]. Mice were housed in an air-conditioned room at 21 ± 1 °C with a lighting period comprised of a 12:12 h light–dark cycle (lights on at 06:30 am). Animals had ad libitum access to water and food (1328 P, Altromin, Lage, Germany) with a content of 11% fat, 24% protein, and 65% carbohydrates with total metabolizable energy of 13.5 kJ/g. At the age of about 7 weeks, female NZO and NMRI mice were mated overnight and fertilization was confirmed by the presence of copulatory plugs the following morning. This day was denoted as 0.5 days post coitum and mice were studied at d14.5 of gestation at the age of about 9–10 weeks. The preconceptionally examined control group was the same age as the pregnant one. These animals were used for all performed tissue isolation, perfusion, and plasma-related experiments as well as histological analysis.

### 4.2. Chemicals

5-HT hydrochloride, HTR antagonists Ketanserin, SB206553, Collagenase Type IV, Collagenase P (Roche), formic acid (puriss. p.a.), and Dexamethasone were obtained from Sigma-Aldrich (Steinheim, Germany). Fura-2 LeakRes (AM) was purchased from Teflabs (Austin, TX, USA), William’s Medium E cell culture media from PAN Biotech (Aidenbach, Germany), and FCS from Gibco (Life Technologies, Darmstadt, Germany). Furthermore, RIPA Lysis Buffer was obtained from Thermo Scientific (Waltham, MA, USA), insulin (Insuman Basal) from Sanofi (Frankfurt, Germany), acetonitrile (HPLC-MS grade) from Wicom (Heppenheim, Germany), and 5-HT-d4 (hydrochloride) from Cayman Chemicals (Ann Arbor, MI, USA). All other reagents were purchased from Merck (Darmstadt, Germany), Carl Roth (Karlsruhe, Germany), or PanReac AppliChem (Darmstadt, Germany). 

### 4.3. Plasma 5-HT Concentration

Whole blood was collected by cardiac puncture after deep isoflurane anaesthesia followed by cervical dislocation. Plasma was obtained by centrifugation (2500× *g*, 10 min, 4 °C) and samples were stored at −80 °C before further analysis. The plasma 5-HT concentrations were determined by Serotonin Research ELISA (LDN, Nordhorn, Germany) according to manufacturer’s instruction.

### 4.4. Total Pancreatic 5-HT Content

After collection, total pancreas was snap frozen in liquid nitrogen and stored at −80 °C until further processing. Tissues were homogenized in ice-cold buffer (0.01 mol/L HCl, 0.1% (*w*/*v*) ascorbic acid, 1 mmol/L EDTA, 4 mmol/L sodium metabisulfite) with 80 s sonication and incubated overnight at 4 °C. Samples were centrifuged (5000× *g*, 15 min, 4 °C) and supernatants were stored at −20 °C until further analysis. The total pancreatic 5-HT content was determined directly after sample centrifugation (2000× *g*, 2 min, room temperature) by an isotopic dilution assay using HPLC-ESI-MS/MS analysis (Method S1).

### 4.5. Islet 5-HT Content and Hormone Secretion

Islets of Langerhans were isolated with the collagenase digestion technique (Collagenase P; 0.5 mg/mL) and hand-picked under the stereomicroscope in the HEPES-buffered Krebs–Ringer medium (115 mM NaCl, 4.7 mM KCl, 2.6 mM CaCl_2_, 1.2 mM KH_2_PO_4_, 1.2 mM MgSO_4_, 20 mM NaHCO_3_, 10 mM HEPES, and 2 mg/mL BSA) saturated with 95% O_2_ and 5% CO_2_ containing 5 mM glucose (for details see Grupe et al. [33]). For each experimental set, 10 freshly isolated islets were preincubated in Krebs–Ringer medium for 1 h containing 5 mM glucose. This was followed by a 1 h incubation with HEPES-buffered Krebs–Ringer medium containing 5, 20, or 20 mM glucose in combination with 10, 100, or 1000 nM 5-HT. The medium was collected and stored at −20 °C until further analysis. To determine the 5-HT content, islets incubated with 20 mM glucose were hand-picked, homogenized in ice-cold buffer (0.01 mol/L HCl, 0.1% (*w*/*v*) ascorbic acid, 4 mmol/L sodium metabisulfite) with 90 s sonication, and incubated overnight at 4 °C. Samples were centrifuged (5000× *g*, 15 min, 4 °C) and supernatants were stored at −20 °C before further analysis. Hormone concentrations were determined by Serotonin Research ELISA (LDN, Nordhorn, Germany), Glucagon ELISA (Mercodia, Uppsala, Sweden), and Rat Insulin ELISA (Mercodia, Uppsala, Sweden) according to manufacturer’s instruction.

### 4.6. Islet Tph2 Gene Expression Analysis

RNA extraction was performed after islet isolation as described previously [77]. Real-time PCR (RT-qPCR) was carried out using an Applied Biosystems 7500 Fast Real-time PCR system (Thermo Fisher Scientific, Waltham, USA) and a QuantiNova SYBR^®^ Green PCR kit (Qiagen, Hilden, Germany). The measurement was performed with a 7500 Fast Real Time PCR instrument (Applied Biosystems, Waltham, USA). Peptidylprolyl isomerase A (*Ppia*) served as an endogenous control and analysis was carried out using the 2^−ΔCT^ method. *Tph2* (NM_173391.3) primer sequences for the forward and reverse primers had been constructed using the PrimerBank (Table 1) [78]:

### 4.7. Immunofluorescence Staining of Pancreatic Sections

After collection, pancreatic tissues were fixed in 4% phosphate-buffered formaldehyde for 24 h. Fixed tissues were then embedded in paraffin according to standard procedures [79]. Representative pancreatic slices (4 µm) were prepared and rehydrated before double immunofluorescence staining was carried out, using mouse monoclonal anti-insulin antibody (1:100,000; Sigma-Aldrich, Steinheim, Germany) and rabbit polyclonal anti-5-HT antibody (1:500; ImmunoStar, Hudson, NY, USA). Primary antibodies were detected with fluorophore-labeled secondary antibodies using Rhodamine Red-X goat anti-mouse (1:200; Jackson ImmunoResearch, West Grove, PA, USA) and Alexa Fluor488 goat anti-rabbit (1:400; Jackson ImmunoResearch, West Grove, PA, USA) as well as DAPI (1:1000; KPL, Gaithersburg, MD, USA). Images were recorded with an upright microscope Eclipse Ni-E (Nikon, Düsseldorf, Germany), equipped with a DS-Fi3 Color Camera (Nikon, Düsseldorf, Germany) and analysis software NIS elements AR 5 (Nikon, Düsseldorf, Germany).

### 4.8. Microfluorimetric Measurement of the ([Ca^2+^]_i_)

Freshly isolated islets of Langerhans were incubated in HEPES-buffered Krebs–Ringer medium containing 5 mM glucose and 2 µM Fura-2 LeakRes (AM) for 45 min at 37 °C. Ten islets were then placed in a perifusion chamber tempered to 37 °C on the stage of an Axiovert 135 microscope (Zeiss, Jena, Germany) equipped with a Fluar (10×, 0.5 N.A.) objective (Zeiss) and perifused with HEPES-buffered Krebs–Ringer medium containing different concentrations of glucose (5 and 20 mM) and 1000 nM 5-HT. Ca^2+^ records were obtained by imaging the fluorescence in the whole islet (excitation at 340 or 380 nm, dichroic separation at 400 nm, emission 510 ± 40 nm bandpass). The fluorescence was recorded using a cooled CCD camera (Pursuit, Diagnostics Instruments, Sterling Heights, USA) and analyzed with Visiview software (Visitron, Munich, Germany). Changes in fluorescence are expressed as the ratio of fluorescence at 340 nm and 380 nm (F_340_/F_380_).

### 4.9. Hepatocyte Isolation and Cell Culture

Hepatocytes were isolated from livers of NZO and NMRI control mice by collagenase digestion technique using a modification of the perfusion method after Baltrusch et al. [80] and studied in short-term primary culture. In short, animals were sacrificed, the peritoneal cavitiy was opened, and a catheter flatly inserted in the portal vein and fixed. The in situ perfusion was executed with a Reglo Analog peristaltic pump (Ismatec, Wertheim, Germany) under continuous flow and the digestion process was performed at 37 °C for 8 to 10 min with Collagenase Type IV. The vena cava was cut to avoid pressure increase within the liver. When liver structure was disrupted, the capsule including the gall bladder was removed and the gathered cells were kept on ice. The isolated cells were filtered and purified and afterwards suspended in supplemented William’s Medium E (5% FCS, 1% penicillin-streptomycin, 5 mM glutamine (2.5%), 0.5% insulin, 0.1 µM dexamethasone (0.01%) and 11 mM glucose). Cell viability was tested by trypan blue exclusion and thereafter cells were seeded on collagen-coated plastic cell culture dishes (Sarstedt, Nümbrecht, Germany) at the density required in the single experiment and incubated in a humidified atmosphere at 37 °C, 95% O_2_, and 5% CO_2_. A total of 0.5 × 10^6^ cells per well were seeded on 6-well plates for glucose production, AKT activation, glycogen and intracellular cAMP content. For glucose uptake, 5000 cells per well were seeded on 96-well plates. After 3–4 h attachment phase, non-adherent cells were removed and the cell culture medium was changed.

### 4.10. Treatment of Primary Hepatocytes

Hepatocytes were cultured overnight (8 h) with indicated stimulants in serum-free William’s Medium E and dexamethasone before experiments were conducted. For stimulation, 1 nM insulin in the glucose production assay and 100 nM insulin in all other experiments were used; 1 µM Ketanserin, 1 µM SB206553, and 0.5 µM 5-HT. HTR antagonists Ketanserin (HTR2A) and SB206553 (HTR2B/C) were applied 30 min prior to the 5-HT stimulus. All experiments were performed within 48 h after plating.

### 4.11. Glucose Production

Primary hepatocytes were washed with PBS and incubated in a glucose-free DMEM starvation medium (1% HEPES, 2% BSA, 44 mM NaHCO_3_, 2.5% glutamine and 1% penicillin-streptomycin) for 8 h. Hepatocytes were provided with a supplemented glucose production DMEM medium (without phenol red, 2 mM sodium pyruvate and 20 mM sodium L-lactate as gluconeogenic substrates) with indicated stimulants and glucose was measured by fluorometric hexokinase glucose assay (abcam, Cambridge, UK) according to the manufacturer’s instructions over 8 h. Assay results were normalized for protein content using a BCA protein assay kit (Sigma-Aldrich, Steinheim, Germany).

### 4.12. Glucose Uptake

Primary hepatocytes were starved in a Krebs–Ringer-phosphate-HEPES (KRPH) buffer containing 20 mM Hepes, 5 mM KH_2_PO_4_, 1 mM MgSO_4_, 1 mM CaCl_2_, 136 mM NaCl, 4.7 mM KCl, and 2% BSA for 40 min supplemented with the indicated stimulants. Afterwards, the medium was removed and a fluorometric 2-DG glucose uptake assay (abcam, Cambridge, UK) was performed according to the manufacturer’s instructions. Briefly, cells were stimulated with 100 nM insulin and the indicated stimulant for 20 min; then, 2-DG was added and cells were incubated for an additional 20 min at 37 °C. The hepatocytes were washed with PBS to remove exogenous 2-DG and to terminate the reaction. Afterwards, cells were lysed, freeze-thawed, and heated at 85 °C for 20 min and the assay was conducted. Assay results were normalized for protein content using a BCA protein assay kit (Sigma-Aldrich, Steinheim, Germany).

### 4.13. Glycogen Content

Primary hepatocytes were washed with phosphate-buffered saline (PBS) three times and then incubated with William’s Medium E without glucose and pyruvate for 2 h to deplete glycogen. Afterwards, cells were incubated with William’s Medium E plus 25 mM glucose and 100 nM insulin supplemented with the indicated stimulant for an additional 2 h. Afterwards, the cells were washed with cold saline and incubated with 30% KOH for 30 min. The cells were scraped and incubated at 95 °C for 20 min. The lysate was then cooled and mixed with absolute ethanol and kept at 4 °C overnight. The cellular glycogen was precipitated by centrifugation (15,000× *g*, 10 min, 4 °C). The supernatant was discarded, the pellet (glycogen fraction) was suspended in water, and the glycogen content was determined with a fluorometric glucoamylase assay kit (abcam, Cambridge, UK) according to the manufacturer’s instructions. Assay results were normalized for protein content using a BCA protein assay kit (Sigma-Aldrich, Steinheim, Germany).

### 4.14. Measurement of Intracellular cAMP

Primary hepatocytes were treated with 500 µM 3-isobutyl-1-methylxanthine (IBMX) and stimulant 30 min prior to further processing to accumulate cAMP in cells. The cells were washed with PBS and treated with 0.1 M HCl for 10 min at room temperature. Successful lysis was ensured by microscopic inspection. Centrifugation (2800× *g*, 3 min) was performed to remove cellular debris. Supernatants were assayed immediately according to a method of Johanns et al. [81]. Therefore, the content was determined using the direct cAMP ELISA kit (Enzo Life Sciences, Farmingdale, NY, USA) according to the manufacturer’s instructions in the acetylated format to increase assay sensitivity. Assay results were normalized for protein content using a BCA protein assay kit (Sigma-Aldrich, Steinheim, Germany).

### 4.15. Measurement of AKT Activation

Primary hepatocytes were stimulated with the indicated stimulant for 3 h and then 100 nM insulin and stimulant were added 10 min before procession in serum-free William’s Medium E. Lysates were prepared in a modified RIPA buffer (150 mM NaCl, 50 mM Tris, pH 7.6, 1% Triton X-100, 0.5% sodium deoxycholate and 0.1% SDS) supplemented with the cOmplete protease inhibitor^TM^ (Roche, Mannheim, Germany) and protein phosphatase inhibitor set (Sigma-Aldrich, Steinheim, Germany) described by Titchenell et al. [82]. Successful lysis was ensured with microscopic inspection. Supernatants were extracted from cellular debris following centrifugation (15,000× *g*, 10 min). Thereafter, AKT activation was assessed with a Phospho-AKT (pSer473)/pan-AKT ELISA kit (Sigma-Aldrich, Steinheim, Germany) according to the manufacturer’s instructions and protein content was assessed using a BCA protein assay kit (Sigma-Aldrich, Steinheim, Germany).

### 4.16. Shared Control Groups

Since the ex vivo primary hepatocyte culture experiments in this study were part of a series of experiments with different stimulants, the same experimental protocols were used in accordance with the 3R principles [83,84]. In addition, according to Törnqvist et al. to reduce the number of animals several stimulation experiments were performed in parallel in order to require only one control group [85]. Where applicable, the control groups of glucose production, glucose uptake and glycogen content, as well as intracellular cAMP content and AKT activation, were shared and may be used by our research group in other publications. The corresponding author for these publications is in all cases Dr. Stephan Scherneck.

### 4.17. Statistics

Statistical calculations and graphical presentation were performed by GraphPad Prism 8 and 9 (GraphPad, La Jolla, San Diego, CA, USA) software. Outliers were identified and removed using the ROUT method. This method was used for the following parameters: Figure 3A–D: insulin and glucagon secretion after static incubation. Data are presented as means ± SEM. For data analysis, non-parametric statistics were applied. To compare means of two mouse strains separately for each of the time points or incubation conditions and to compare differences within one strain over the period of time or between different incubation settings (e.g., NMRI preconceptional vs. d14.5 or 5 mM vs. 20 mM glucose), Mann–Whitney U test was applied. To analyze alterations of more than two independent samples within two strains over the period of time, a Kruskal–Wallis H test followed by a Dunn’s multiple-comparison test was applied. For further multiple group comparisons of different stimulation groups, a two-way ANOVA was used. Differences were considered significant if *p* < 0.05. *p* values were indicated as * *p* < 0.05, ** *p* < 0.01, *** *p* < 0.001 and **** *p* < 0.0001.

### 4.18. Data Availability

The data that support the findings of this study are available from the corresponding authors upon reasonable request.

## Figures and Tables

**Table 1:** Primers for RT-qPCR.

Gene	Fwd	Rev
*Tph2*	5′-gcaagacagcggtagtgttct-3′	5′-cagtccacgaagatttcgactt-3′

## Data Availability

All data presented in this study are available on request from the corresponding author.

## Supplementary Materials

The following supporting information can be downloaded at: https://www.mdpi.com/article/10.3390/ijms23126434/s1.
