# Supplementary material for: Role of Serotonin (5-HT) in GDM Prediction Considering Islet and Liver Interplay in Prediabetic Mice during Gestation
Source: Int J Mol Sci. 2022 Jun 9;23(12):6434. doi: 10.3390/ijms23126434 (PMC9224346; doi:10.3390/ijms23126434)
Supplement: Supplementary file 1 [file ijms-23-06434-s001.zip › ijms-1757957-supplementary.pdf]

Supplementary Materials: Role of serotonin (5-HT) in GDM Prediction Considering Islet and Liver Interplay in Prediabetic Mice during Gestation

Melissa Asuaje Pfeifer [1†,], Moritz Liebmann [1†,], Till Beuerle [2,], Katharina Grupe [1] and Stephan Scherneck [1,]

1 Institute of Pharmacology, Toxicology and Clinical Pharmacy, Technische Universität Braunschweig, Mendelssohnstraße 1, D-38106 Braunschweig, Germany; melissa.asuaje-pfeifer@tu-braunschweig.de (M.A.P.); m.liebmann@tu-braunschweig.de (M.L.); k.grupe@tu-braunschweig.de (K.G.)

2 Institute of Pharmaceutical Biology, Technische Universität Braunschweig, Mendelssohnstraße 1, D-38106 Braunschweig, Germany; t.beuerle@tu-braunschweig.de

* Correspondence: s.scherneck@tu-braunschweig.de; Tel.: +49-531-391-8440

† These authors contributed equally to this work.

Method S1. Total pancreatic 5-HT content. Analysis was performed using a HPLC (Agilent 1200 Series, Agilent Technologies, Waldbronn, Germany) in combination with ESI-MS/MS detection (Mass Spectrometer 3200 QTrap, Applied Biosystems MDS Sciex, Darmstadt, Germany). The chromatographic separation was performed on a Raptor Biphenyl column (150 mm x 2.1 mm, particle size 2.7 µm) purchased from (Restek, Bad Homburg, Germany) including a 5 mm pre-column cartridge of the same material. The following gradient was applied at a flow rate of 250 µL/min (solvent A: 0.3% formic acid in water, solvent B: 0.3% formic acid in acetonitrile): 0 - 2 min (95% A), 2 - 8 min (95 - 35% A), 8 - 9 min (35% A), 9 - 10 min (35 - 95% A), 10 - 18 min (95% A). Ionization and fragmentation was performed in ESI-positive mode and quantification was achieved in the multi reaction monitoring (MRM) mode using the following MS-parameters. All gases were nitrogen. Curtain gas: 50 psi, ionization voltage: 5500 V, ion source temperature: 500 °C, gas 1: 50 psi, gas 2: 50 psi, collision-activated dissociation (CAD) gas: high, dwell time for all transmissions was set to 100 ms each. Three transitions per analyte and internal standard (IST) 5-HT-d4 (hydrochloride) were selected.

Quantification was achieved by an isotope dilution assay method. 5-HT content was calculated by direct comparison of the corresponding quantifier transitions for the analyte (177.0 → 160.0) and IST (181.0 → 164.0), respectively. Data analysis and integration was achieved with Analyst 1.6.2 Software (Applied Biosystems MDS Sciex, Darmstadt, Germany). Factors were taken into account to compensate the hydrochloride and deuterated nature of the IST and the sample dilution by addition of the IST. To confirm peak identities, the presence and ratios of the corresponding quantifiers/qualifier transition signals were monitored and taken into account as well. To exclude possible carry-over effects, blank determinations by injecting pure methanol were performed at regular intervals during the analysis sequences. Limit of detection (LoD), limit of quantification (LoQ) and linear analytical range were determined experimentally using 5-HT-d4 (hydrochloride) spiked to the original sample matrix. Linearity was tested in the range of 0, 0.2, 1, 5, 10, 25, 50, 100, 200, 400.

Table S1. Ion transitions and compound specific MS/MS parameters for the detection of 5-HT and 5-HT-d4 (IST). Quantifier transitions in bold. Internal standard (IST), declustering potential (DP), cell entrance potential (CEP), entrance potential (EP), collision energy (CE), cell exit potential (CXP).

	Transitions [m/z]	DP [V]	CEP [V]	EP [V]	CE [eV]	CXP [V]
**Analyte**	**177.0 → 160.0**	10.0	12.0	4.0	15.0	4.0
5-HT	177.0 → 132.0	21.0	12.0	4.0	29.0	4.0

	177.0 → 115.0	21.0	12.0	4.0	37.0	5.0
**IST**						
5-HT-d4	**181.0 → 164.0**	10.0	12.0	4.0	15.0	4.0
	181.0 → 136.0	21.0	12.0	4.0	29.0	4.0
	181.0 → 118.0	21.0	12.0	4.0	37.0	5.0

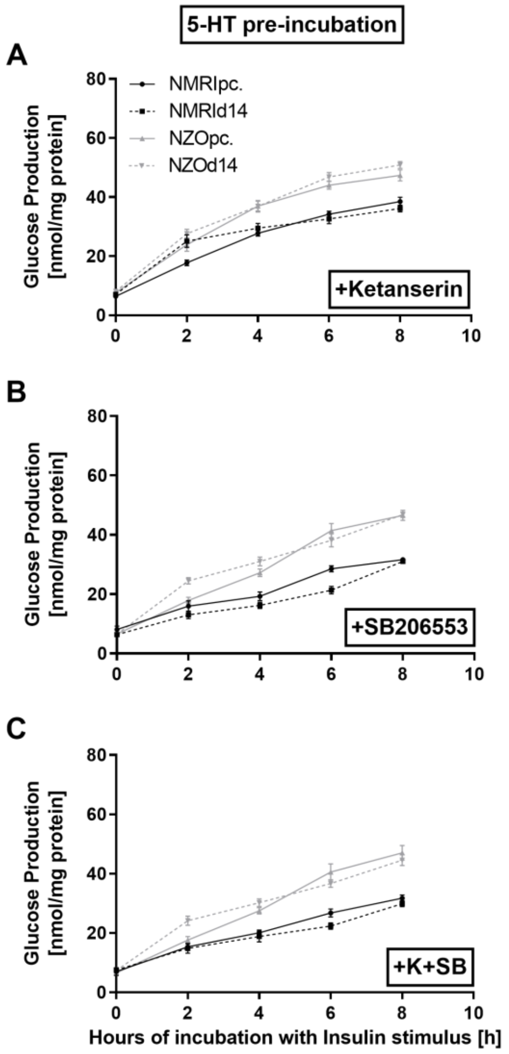

Figure S1. Effects of HTR antagonists on hepatic glucose utilization in primary hepatocytes. Glucose production in cultured primary hepatocytes of NZO and NMRI control mice at time points preconceptional and d14.5 of gestation. Preincubation with 5-HT (**A, B, C**) and stimulation with (**A**) Ketanserin (HTR2A antagonist), (**B**) SB206553 (HTR2B/C antagonist) or (**C**) co-stimulation with both antagonists (KSB). Data are presented as means ± SEM (n = 5 animals per group).

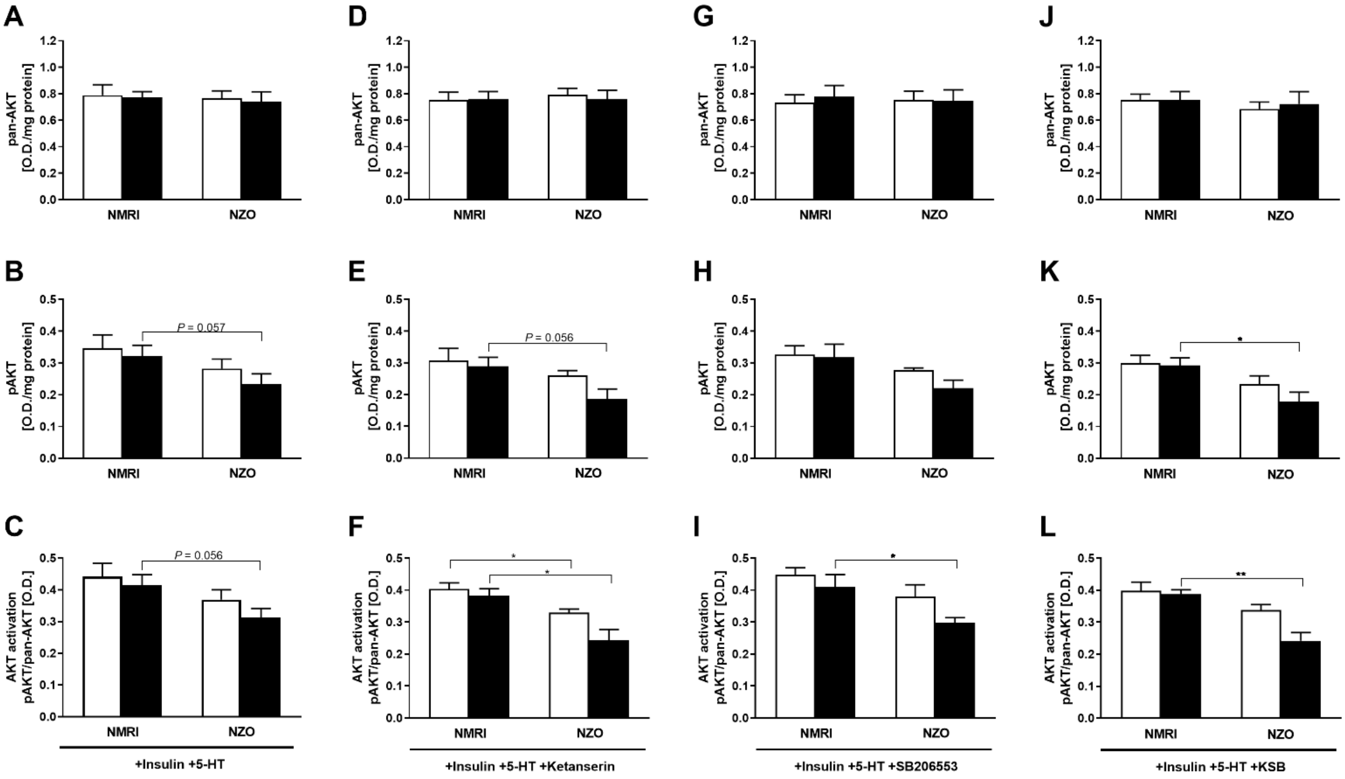

Figure S2. AKT signaling in primary hepatocytes after 5-HT stimulation and administration of HTR antagonists. (**A, D, G, J**) Pan-AKT, (**B, E, H, K**) phospho-AKT and (**C, F, I, L**) the derived pAKT/pan AKT-ratio with insulin, 5-HT stimulation and co-incubation with Ketanserin (HTR2A antagonist) and SB206553 (HTR2B/C antagonist) in primary hepatocytes of NZO and NMRI mice (at time points preconceptional (white bars) and d14.5 of gestation (black bars). Data are presented as means ± SEM (n = 4-6 animals per group). * $p < 0.05$, ** $p < 0.01$.
